# Introducing the 2024 Transcatheter Cardiovascular Therapeutics (TCT) Shark Tank Presentations

**DOI:** 10.1016/j.jacbts.2024.11.002

**Published:** 2024-12-23

**Authors:** Douglas L. Mann

The editors of *JACC: Basic to Translational Science* are pleased to feature 3 outstanding Shark Tank presentations from the 2024 Transcatheter Cardiovascular Therapeutics (TCT) meeting, held in Washington, DC, from October 27 to 30, 2024. The TCT is one of the most significant global meetings dedicated to interventional cardiovascular medicine organized by the New York-based Cardiovascular Research Foundation. Since its inception, the TCT has served as a premier platform for cardiovascular medical device innovation, spotlighting pivotal shifts and promising advancements that shape the future of the field. Over the past decade, the TCT’s scientific program has featured a dedicated Innovation Program track, showcasing the latest technological breakthroughs in cardiovascular medicine. This track also facilitates broader discussions on the global innovation landscape, including its challenges and opportunities. Since 2004, the Innovation Program's highlight has been a special session aimed at promoting the development of emerging technologies poised to revolutionize the diagnosis and treatment of cardiovascular disease. This session, designed as a public competition, draws inspiration from the popular television show “Shark Tank,” where startups pitch their ideas to seasoned investors, seeking their guidance and mentorship. The annual Shark Tank presentations at TCT are evaluated by an experienced panel of multidisciplinary experts who evaluate the entrants on the 6 established criteria for the competition: unmet clinical need, technology differentiation, intellectual property position/viability, biological proof of concept, regulatory pathway, and commercialization potential. This is the second consecutive year that *JACC: Basic to Translational Science* has collaborated with the Cardiovascular Research Foundation to shine a light on exciting new cardiovascular therapeutics.

This issue of *JACC: Basic to Translational Science* features research letters describing 3 of 6 TCT Shark Tank finalists for the year 2023, along with video links to their presentations and the discussions by the judges. Here, we provide brief summaries of 3 of the companies whose work is featured in this issue of *JACC: Basic to Translational Science.* These companies represent a very diverse portfolio of technologies, each of them trying to address a different unmet need through innovative solutions.

Symbiosis (https://www.symbiosis-cm.com/) is advancing the development of an adjustable transcatheter mitral valve replacement system known as the Symbiosis ValSync. This innovative transcatheter mitral valve replacement system includes 2 balloons made from a flexible, deformable material designed to expand and conform to the surrounding valve anatomy, enabling real-time customization of size, shape, and anchoring to accommodate unique anatomical structures. According to the company, potential advantages of this system include expanded patient eligibility, an improved safety profile, and a streamlined, 3-step procedure that offers a shorter learning curve for clinicians. The editors would like to congratulate Symbiosis on being the winner of this year's TCT Shark Tank presentation!

StarTric (https://www.startric.it/) is developing a percutaneous transcatheter technology aimed at replicating the Clover open heart technique for repairing the tricuspid valve. The StarTric TriClover device is an innovative, next-generation, triple edge-to-edge transcatheter repair technology designed to address the limitations of first-generation transcatheter tricuspid valve solutions. It aims to restore coaptation in patients with tricuspid regurgitation by reliably creating a “Clover” configuration similar to that achieved through open surgical repair. Specifically developed for the tricuspid valve, the Clover edge-to-edge repair technique has demonstrated excellent and durable outcomes in complex cases of tricuspid valve regurgitation.

Pumpinheart (https://pumpinheart.com/) is a spinout company from the Royal College of Surgeons in Ireland that is developing a device to modulate left atrial pressure to relieve the symptoms of heart failure with a preserved ejection fraction. Pumpinheart’s miniature implantable left atrial/left ventricular pump (PReduction device) offers a novel therapeutic approach, operating by moving a precise volume of blood from the left atrium, across the mitral valve, into the left ventricle during diastole to lower left atrial pressure and alleviate symptoms. The PReduction device is designed as a transfemorally delivered implantable pump and motor that is ECG-gated and powered by a subcutaneously implanted battery pack.

The editors of *JACC: Basic to Translational Science* would like to congratulate the members of the research teams of Symbiosis, StarTric, and Pumpinheart for helping to advance the field of translational cardiovascular medicine. I trust that our readers will share my perspective that we are on the cusp of introducing innovative new strategies that are poised to enhance the treatment outcomes for individuals afflicted with cardiovascular disease. I would be remiss if I failed to express my gratitude to Drs. Juan Granada and Gregory Kaluza, alongside our editorial team, for ensuring the timely publication of this special issue just a month following the TCT conference. My thanks extend to Stephanie Gutch, Senior Director of Digital at the Cardiovascular Research Foundation, for her invaluable assistance in integrating video content from the TCT conference onto the *JACC* website. My appreciation also goes out to the exceptional *JACC* team for making this publication a reality, including, but not limited to, Justine Turco (Divisional Vice President, Scientific Publications and Guidelines, American College of Cardiology) and Monica Payne-Emerson (Executive Managing Editor for the *JACC* journals), with a special personal thanks to Kim Trevey (Managing Editor) and Jennifer Rapp (Editorial Assistant), whose remarkable efficiency and commitment to publishing the Shark Tank issues are not only remarkable but truly indispensable.

As always, I look forward to receiving your comments on this special edition of the Journal, whether it be through social media using the hashtag #JACC: BTS or via e-mail at jaccbts@acc.org.

